# Automatic Artifact Detection Algorithm in Fetal MRI

**DOI:** 10.3389/frai.2022.861791

**Published:** 2022-06-16

**Authors:** Adam Lim, Justin Lo, Matthias W. Wagner, Birgit Ertl-Wagner, Dafna Sussman

**Affiliations:** ^1^Department of Electrical, Computer and Biomedical Engineering, Faculty of Engineering and Architectural Sciences, Toronto Metropolitan University, Toronto, ON, Canada; ^2^Institute for Biomedical Engineering, Science and Technology (iBEST), Toronto Metropolitan University and St. Michael's Hospital, Toronto, ON, Canada; ^3^Division of Neuroradiology, The Hospital for Sick Children, Toronto, ON, Canada; ^4^Department of Medical Imaging, Faculty of Medicine, University of Toronto, Toronto, ON, Canada; ^5^Department of Obstetrics and Gynecology, Faculty of Medicine, University of Toronto, Toronto, ON, Canada

**Keywords:** deep learning, fetal MRI, convolutional neural networks, image classification, imaging artifacts

## Abstract

Fetal MR imaging is subject to artifacts including motion, chemical shift, and radiofrequency artifacts. Currently, such artifacts are detected by the MRI operator, a process which is subjective, time consuming, and prone to errors. We propose a novel algorithm, RISE-Net, that can consistently, automatically, and objectively detect artifacts in 3D fetal MRI. It makes use of a CNN ensemble approach where the first CNN aims to identify and classify any artifacts in the image, and the second CNN uses regression to determine the severity of the detected artifacts. The main mechanism in RISE-Net is the stacked Residual, Inception, Squeeze and Excitation (RISE) blocks. This classification network achieved an accuracy of 90.34% and a F1 score of 90.39% and outperformed other state-of-the-art architectures, such as VGG-16, Inception, ResNet-50, ReNet-Inception, SE-ResNet, and SE-Inception. The severity regression network had an MSE of 0.083 across all classes. The presented algorithm facilitates rapid and accurate fetal MRI quality assurance that can be implemented into clinical use.

## Introduction

### Background

Fetal MRI artifacts can severely degrade image quality and the radiologist's ability to use the image for diagnostic decision-making. Detection of such artifacts is currently done visually by the MRI operator, a process which is subjective, time consuming, and prone to errors. This is ultimately an intensive quality control task for the interpreting physician. Automating this process immediately alerts the MRI operator after sequence acquisition whether the images are usable for diagnostics or whether the sequence will need to be repeated, thus alleviating the cognitive burden, and improving diagnostic accuracy. Such automatic detection of imaging artifacts can be accomplished using machine learning methods.

### Related Works

Prior approaches to automatic detection of imaging artifacts and evaluation of overall quality include using gaussian naïve bayes, support vector machines, and random forests machine learning techniques (Sujit et al., 2018). While sometimes reliable, these classifiers are limited by the manually selected features fed into them. This process makes these classifiers a sub-optimal solution because the performance of a classifier is heavily dependent on the feature selection procedure. A more efficient and modern approach incorporates deep learning and, in particular, convolutional neural networks (CNN). CNN architectures have been shown to accurately classify medical images mainly because of their automatic feature detection element. An example of this approach was recently implemented using MRIs of the brain which were classified into “motion-free” vs. “motion-corrupted” (Fantini et al., 2021). While this method achieved good results with an overall accuracy of 86.3%, it did not account for the type of artifact present, which can be important for many applications. Another study used a similar CNN approach and aimed to classify abdominal MRIs into 3 different classes: “poor,” “diagnostic,” and “excellent” (Ma et al., 2020). The authors reported an accuracy of 65%, but when converted to a binary problem by combining the “diagnostic” and “excellent” classes, an accuracy of 84% was achieved. They concluded that multi-class classification problems are more difficult to train in comparison to binary problems due to similarities within the dataset.

Currently, other researchers have found successes in this domain, but are constrained by the number of artifacts classified, and/or are not generalizable to full body anatomy. Most existing automatic artifact detection algorithms are designed to identify singular artifact types such as motion. For example, Küstener et al. incorporated a method to spatially recognize and quantify motion artifacts in MR images pertaining to the head and abdominal regions (Küstner et al., 2018). While capable of achieving an average accuracy of 86%, it does not alleviate the time consuming and cumbersome aspect in the clinical setting as it still requires manual inspection of other artifacts to ensure diagnostic usability. Other methods aim to execute similar solutions but are idealistic in practice due to related reasons. However, Gagoski et al. proposed a CNN to detect fetal motion in half-Fourier single-shot fast spin echo (HASTE) sequences (Gagoski et al., 2022). While the algorithm was limited to one type of artifact, they also added a motion correction feature which would be part of the future development of our proposed project. In our algorithm, we address a wider series of artifacts not studied in the Gagoski solution. Furthermore, other artifact detection approaches are anatomy specific, therefore restricting their usability to explicit scenarios. The majority are mainly focused on the head region such as in Oksuz (2021) and are not generalizable to the full body.

Our approach makes use of CNNs which have made significant improvements across varying problems such as classification, segmentation, and object detection. Recently, CNN architectures have been further advanced in terms of accuracy and training speed. Two examples are ResNet-50 and Inception which address the vanishing gradient problem and reduce computational resources, respectively (Khan et al., 2020). In addition, squeeze and excitation blocks have gained recognition as they have shown to improve results with minimal computation cost (Roy et al., 2019). Other authors have implemented hybrid networks containing the different modules, namely, ResNet-Inception, SE-ResNet, and SE-Inception. Szegedy et al. stated that combining skip connections from ResNet with the Inception network accelerated network training significantly and provided greater performance in some instances (Szegedy et al., 2016). Furthermore, Hu et al. performed experiments involving the addition of SE blocks to benchmark networks such as ResNet and Inception (Hu et al., 2019). They found that across varying datasets, the addition of SE significantly improved network performance in comparison to their standalone counterparts.

### Motivations and Contributions

In our study, we propose a novel algorithm to detect and classify artifacts on full body fetal MR imaging using four different classes of artifacts: (1) motion, (2) chemical shift, (3) radio-frequency, and (4) no artifacts. We aim to fill in the gaps in this domain by developing a deep learning algorithm that can detect and score a variety of artifacts and can be summarized for full anatomy scans. Specifically, the algorithm makes use of a CNN ensemble approach where the first CNN aims to identify and classify any artifacts in the image, and the second CNN intends to determine the severity of artifacts detected. With the recent success of multi-model CNNs, particularly skip connections, inceptions modules, and SE blocks, we intend to incorporate these components in our proposed algorithm as they have been proven to be beneficial in similar computer vision tasks. This algorithm will be a useful quality control tool for radiologists and improve imaging and diagnostic protocols in a clinical setting.

### Paper Outline

The rest of the paper is organized as: Materials and Methods (section 2), Results (section 3), Discussion (section 4), and Conclusion (section 5). In section Materials and Methods, the dataset used, proposed method, and experiment parameters are covered. Section Results provides the results of the experiments including algorithm performance and an extensive ablation study. Section Discussion contributes an interpretation of the results and relates it to current literature. Section Conclusion summarizes the overall conclusion by defining key findings and future steps.

## Materials and Methods

### Dataset

**Figure 1:**
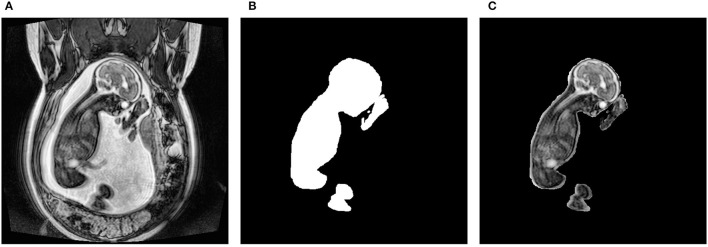
Preprocessing steps to obtain the fetal segmentation. **(A)** represents the original 2D slice. **(B)** shows the manually segmented mask. **(C)** displays the overlay of the mask onto the original slice.

The dataset consisted of 31 anonymized 3-dimensional (3D) fetal MRIs without structural abnormalities that were acquired at the Hospital for Sick Children, Toronto. The dataset and study have been approved by the Research Ethics Board. They were collected using both an SSFP sequence on a 1.5T scanner and a 3D SSFP sequence with SENSE on a 3.0T scanner. The former sequence resulted in a resolution of 384 × 384, while the latter was 512 × 512. All scans were extracted in the coronal plane. This resulted in a total of 2,250 2-dimensional (2D) slices. Each slice within a single scan was manually segmented using the Amira-Avizo software (Berlin, Germany). As shown in Figure 1, the resulting masks were made transparent and superimposed onto their corresponding slice so that only the fetus was visible. The resulting images were then saved as PNG files and their pixel intensities were normalized. This type of normalization involves dividing each pixel by the maximum intensity found in the image, resulting in values between 0 and 1. This preprocessing step was necessary since the scans were obtained using different sequences and varying parameters. Following these steps, the dataset was given to 2 pediatric neuroradiologists who manually labeled each slice in a consensus reading. They determined which artifacts were present in each image slice and assigned one of the following severity scores: none, mild, medium, or severe. The categorical labels were converted to numerical values in order to train the networks. Specifically, the artifact types were one-hot-encoded where 1 meant the artifact was present and 0 represented it was not. Furthermore, the severity scores were converted to a discrete range from 0 to 3, where 0, 1, 2, and 3, portrayed none, mild, medium, and severe, respectively.

The 2,250 2D images were then divided into a training, validation, and testing set using an 80:10:10 split, resulting in a training set of 1,800 images, a validation set of 225 images, and a testing set of 225 images. Each image was also resized to 512 × 512 because CNNs can only handle a single input size.

### Architecture

**Figure 2:**
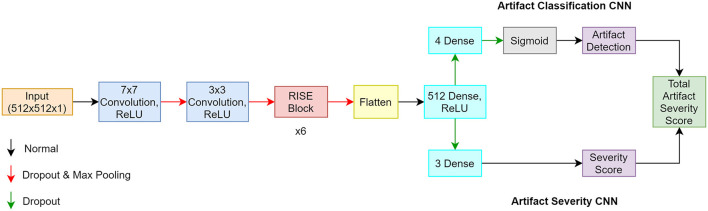
Architecture of proposed algorithm where the classification CNN is the top pathway, and the regression CNN is the bottom pathway.

The proposed artifact classification network uses a CNN framework and takes in our manually segmented fetal MR images to ensure any artifacts detected are directly affecting fetal tissue. The architecture involves components inspired from the Inception, Resnet-50, and Squeeze and Excitation networks. These three elements were combined to create the Resnet-Inception-Squeeze-and-Excitation (RISE) module. The first part of the network is composed of sequential convolution, dropout, and max pooling layers. This is followed by the main part of the network which incorporates 6 consecutive RISE blocks. The last part is composed of 2 dense layers containing 512 and 4 neurons, respectively. The last dense layer contains a sigmoid activation function permitting multi-label classification where more than 1 artifact can exist in a single image. This produced an array containing probabilities pertaining to each artifact class. The artifact severity regression network uses the same architecture as described, but aims to output a severity score between 0 (none) and 3 (severe). The last dense layer was changed to contain 3 neurons and the sigmoid activation function was removed in order to accommodate the new problem. This allowed the network to produce an array where each value represented the severity of each artifact type. A mean square error was also implemented for the loss function. A total artifact severity score was computed by multiplying the outputs from each network. Since the outputs from each network were indexable arrays, we were able to extract each numerical value and multiply them accordingly. This score was representative of the type and severity of artifacts present. Based on the thresholding system seen in Table 1, the algorithm was able to convert the score into text form where the final output was the artifact type (motion, chemical shift, radiofrequency) and the severity level (none, mild, medium, severe). The overall algorithm framework is illustrated in Figure 2.

**Table 1:** Thresholding system for determining artifact grade.

	**Score ** < = **0.5**	**0.5 < Score ** < = **1**	**1 < Score ** < = **2**	**Score **> = **2**
Artifact grade	None	Mild	Medium	Severe

#### Inception Module

**Figure 3:**
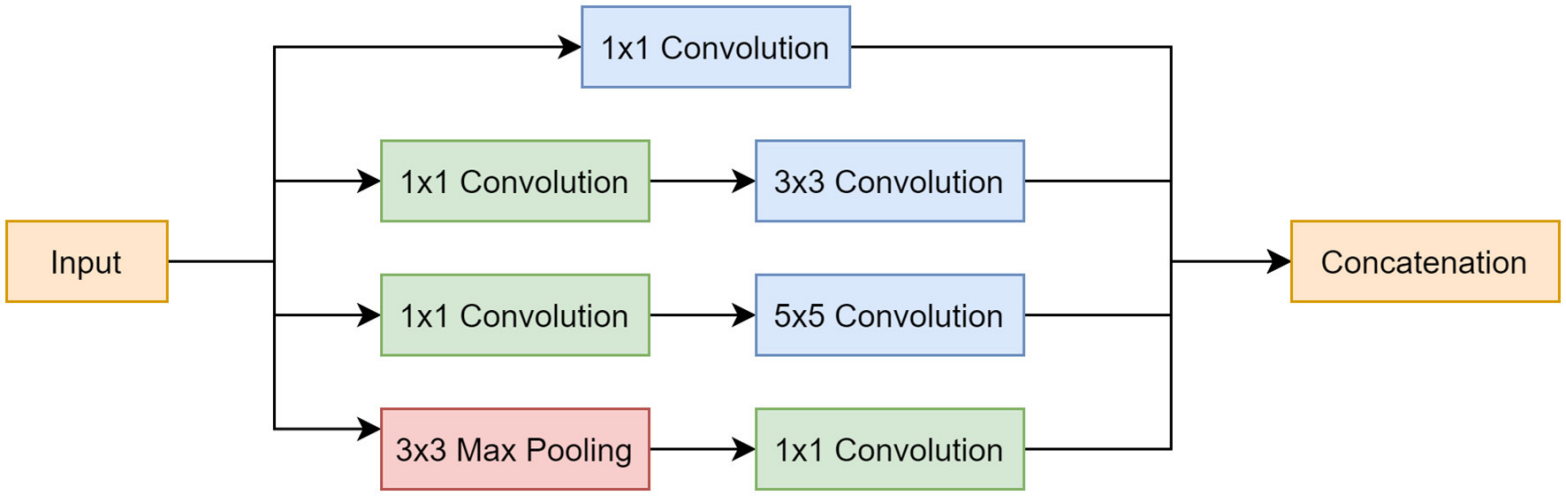
Inception module block diagram. Top pathway follows a 1 × 1 convolution. Second pathway involves a 1 × 1 convolution, followed by a 3 × 3 convolution. Third pathway incorporates a 1 × 1 convolution, followed by a 5 × 5 convolution. Last pathway starts with a 3 × 3 max pooling layer and is followed by a 1 × 1 convolution. All pathways are concatenated at the end.

The Inception module was implemented for 2 main reasons: (1) it makes the network more computationally manageable as it creates a wider network as opposed to a deeper one, and (2) it limits the number of input channels by employing a 1 × 1 convolution layer before the 3 × 3 and 5 × 5 convolutions and after the max pooling layer, which further reduces model complexity. Another reason was that applying different sized filters allowed for the detection of features of varying sizes. This was beneficial for this application as artifacts exist in different sizes, where some are more global and cover most of the image, others are local and only exist in a small portion of the image. The Inception module is shown in Figure 3.

#### ResNet Module

**Figure 4:**
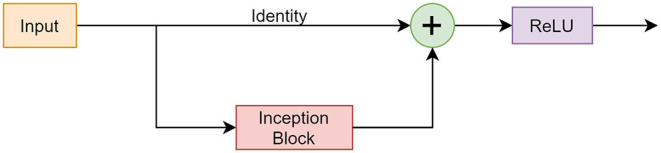
**(A)** standard skip connection is shown. **(B)** implementation of skip connection with Inception module.

The ResNet architecture introduced a skip connection mechanism that is an accepted and beneficial practice used in many CNNs today (Orhan and Pitkow, 2018). This mechanism was implemented to address the vanishing gradient problem which is a common problem during the backpropagation step of training a CNN (Yasrab, 2019). Through backpropagation, the gradient of the loss function with respect to a weight can become infinitely small and, therefore, the weights stop updating. A skip connection combats this by adding the output from a previous layer to the layer ahead. This is shown in Figure 4A where x is the input (identity), and F(x) is the learned features. Figure 4B illustrates the implementation of the skip connection along the Inception module.

#### Squeeze and Excitation Module

**Figure 5:**
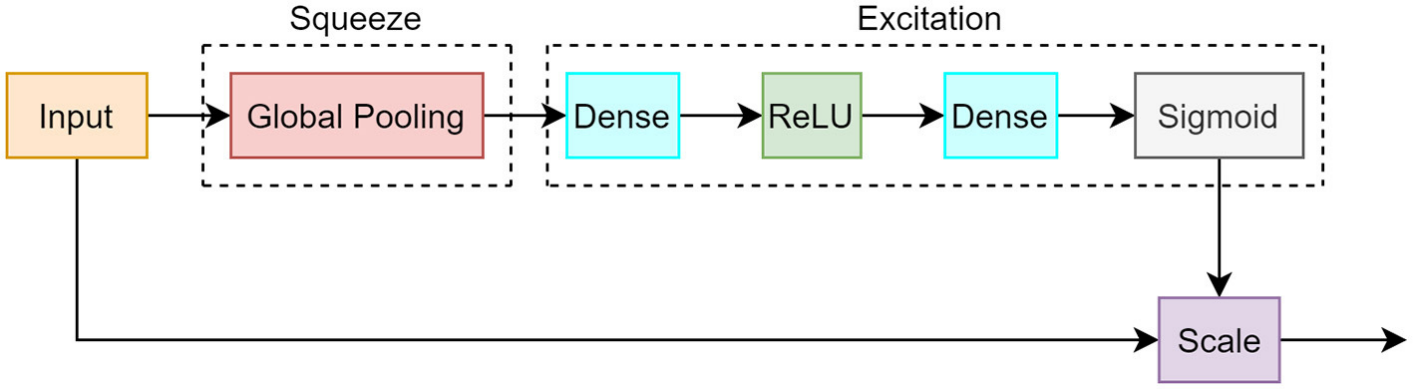
Block diagram of squeeze and excitation module. The squeeze segment consists of a global pooling operation which creates a value for each channel. The excitation component uses dense, ReLU, and sigmoid layers that correspond to the importance of each channel.

The SE module consists of 2 different operations: squeeze and excitation. During the squeeze procedure, a single value is created for each channel of the input by using a global average pooling computation. This is followed by an excitation process that takes the output vector from the squeeze operation and creates a set of weights per channel. Ultimately, a sigmoid activation function is employed to create weight values between 0 and 1, which correspond to how much attention each channel should receive. These weights are multiplied to each corresponding channel in a layer termed as the scale layer. The weighted channels provide greater emphasis on important features and less emphasis on background features. The SE module is illustrated in Figure 5 and the equations are shown in 1.

Equation (1) shows the squeeze operation (*S*_*n*_) per feature map, where *I*_*n*_ is the input feature map, and *H* and *W* are spatial dimensions.


(1) $$\begin{array}{l} S_{n}=F_{Squeeze}\left(I_{n}\right)=\;\frac{1}{H\times W}\overset{H}{\underset{i=1}{\sum }}\overset{W}{\underset{j=1}{\sum }}I_{n}\left(i,j\right) \end{array}$$

Equation (2) illustrates the excitation operation (*E*_*n*_) per feature map, where δ represents the ReLU activation, and *g* is the complexity value which is used for channel parameter reduction and to aid in model generalization. Through experimentation, we found *g* = 8 yielded the best results. σ represents the sigmoid activation.


(2) $$\begin{array}{l} E_{n}{=F}_{Excite}\left(S_{n}\right)=\left(\left(\delta ,\frac{S_{n}}{g}\right),\sigma \right) \end{array}$$

Equation (3) displays the scaling step where the output (*O*_*n*_) is the newly weighted feature map based on the multiplication of the excitation weights and original input feature map.


(3) $$\begin{array}{l} O_{n}=E_{n}\times I_{n} \end{array}$$

#### RISE Module

**Figure 6:**
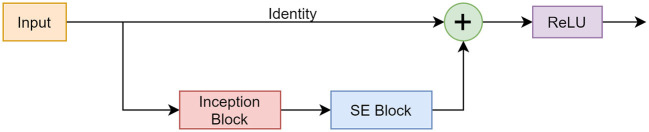
Framework of the RISE module consisting of a skip connection, Inception block, and SE block.

The RISE module incorporated components from the described networks. Figure 6 shows the design and functionality of the block.

### Experiments

The proposed model was trained and validated using the Compute Canada Cedar cluster which is listed for general use. The cluster was set up using 1 node, where each node consisted of 4 NVIDIA V100 Volta (32 GB) GPUs, 2 Intel Silver 4216 Cascade Lake (2.1GHz) CPUs with 32 cores, and a maximum memory of 187 GB.

The networks were compiled using the Keras API with a TensorFlow (Mountain View, California) backend and the coding language used was Python version 3.6.13 (Amsterdam, Netherlands). The built-in Adam optimizer was implemented with the default parameters except the initial learning rate which was changed to 1 × 10^−5^. The Adam optimizer was chosen as opposed to the usual stochastic gradient descent (SGD) optimizer due to its adaptive learning rate which has shown to enhance performance in CNNs (Dogo et al., 2018). The classification model used a sigmoid activation function after the last dense layer which produced probabilities that were between 0 and 1 and were also independent of the other classes. This was implemented as the task was a multi-label classification problem where an image could contain more than one artifact, thus belonging to more than one class. The sigmoid activation function was paired with a binary cross entropy (BCE) loss which similarly is computed for every independent output node. Both classification and regression networks were trained separately for a total of 100 epochs with a batch size of 8. Additionally, a 5-fold cross validation method was used in order to rigorously assess model performance. The networks were deployed together in parallel in order to obtain our final model outputs of artifact type and severity.

The following metrics were monitored for both training and validation phases of the classification network: accuracy (exact match ratio), F1 score, precision, and recall. Accuracy was the strictest metric as the prediction was only considered correct if it exactly matched its corresponding label. F1 score is the harmonic mean between precision and recall, which can give a better representation of model performance in comparison to accuracy. Precision was beneficial to measure per class accuracy. Recall can indicate how well a class is used as a prediction. Calculating the mentioned metrics provided insight on how well the model was performing and what aspects needed improvement.

Below are the equations used, where *n* is the number of images, *I* is the indicator function, *Ytrue* is the ground truth, *Ypred* is the prediction, *TP* is true positives, *FP* is false positives, and *FN* is false negatives.


(4) $$\begin{array}{l} \text{Accuracy }=\frac{1}{n}\overset{n}{\underset{i=1}{\sum }}I\left(Ytrue_{i}\;=Ypred_{i}\right) \end{array}$$

(5) $$\begin{array}{l} \text{Precision }=\;\frac{TP}{TP\;+\;FP} \end{array}$$

(6) $$\begin{array}{l} \text{Recall }=\;\frac{TP}{TP\;+\;FN} \end{array}$$

(7) $$\begin{array}{l} F1\;\text{Score }=\frac{2\left(\text{Precision }\times \;\text{Recall}\right)}{\text{Precision }+\;\text{Recall}} \end{array}$$

For further validation, the proposed architecture was also compared to the most used and well-known CNNs for image classification: VGG-16, Inception, and ResNet-50 (Szegedy et al., 2014; He et al., 2015; Simonyan and Zisserman, 2015). We also compared it with other hybrid models including ResNet-Inception, SE-ResNet, and SE-Inception. These experiments were conducted to check whether the proposed network outperformed pre-existing state-of-the-art models. These networks replaced the main part of the CNN, but the input and output layers were modified to fit this specific application. The hyperparameters remained the same throughout the models, and the same metrics were monitored for each network.

An extensive ablation study was also conducted where different components of the main architecture were varied slightly to determine their contribution to the model performance. Specifically, tests were done where (1) the inception module was omitted and replaced with a standard convolution layer, (2) the skip connections were removed entirely, and (3) the SE blocks were eliminated. This study was carried out to test whether the RISE module is superior to its standalone counterparts.

Since the second CNN, the artifact severity network, followed the same framework as the classification CNN with minor adjustments, mean squared error (MSE) was the only monitored metric. MSE, which is defined below, is a regression metric that is used to calculate the average of squared differences between the ground truth and predicted values. In the equation, *n* represents the number of data points, *Ytrue* is the ground truth, and *Ypred* is the predicted output from the CNN.


(8) $$\begin{array}{l} MSE\;=\frac{1}{n}\overset{n}{\underset{i=1}{\sum }}{\left(Ytrue_{i}-Ypred_{i}\right)}^{2} \end{array}$$

## Results

**Figure 7:**
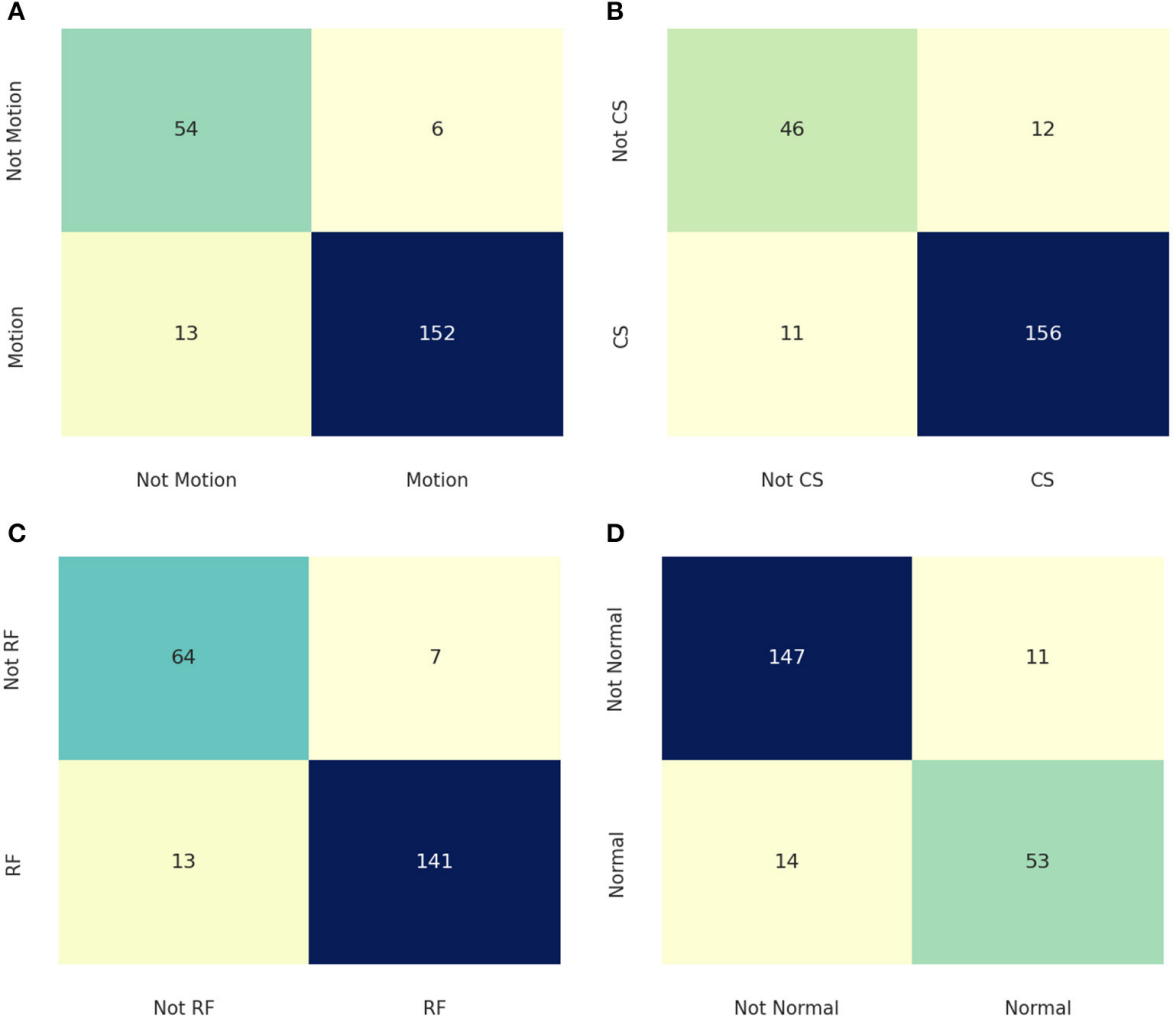
Confusion matrices for the different classes where the ground truth is on the y-axis, and the prediction is on the x-axis. **(A)** is the confusion matrix for the “Motion” class. **(B)** shows the confusion matrix for the “Chemical Shift” class. **(C)** displays the confusion matrix for the “Radio-Frequency” Class. **(D)** illustrates the confusion matrix for the “Normal” class.

The performance of the artifact classification network is shown in Figure 7. This figure shows binary confusion matrices for each class where the ground truth is on the y-axis and the predicted class is on the x-axis. These confusion matrices provide a visual representation of how well the classifier performed on the test dataset by quantifying the true negatives (top left box), false positives (top right box), false negatives (bottom left box), and true positives (bottom right box). These results were then used in computing the accuracy, precision, recall, and F1 score which are summarized in Table 2. For the “Motion” class, the network achieved an accuracy of 91.56%, precision of 96.20%, recall of 92.12%, and F1 score of 94.12%. The “Chemical Shift” class achieved an accuracy of 89.78%, precision of 92.86%, recall of 93.41%, and F1 score of 93.13%. The “Radio-frequency” class attained an accuracy of 91.11%, precision of 95.27%, recall of 91.56%, and F1 score of 93.38%. Lastly, the “Normal” class achieved an accuracy of 88.89%, precision of 82.81%, recall of 79.1%, and F1 score of 80.91%.

**Table 2:** Accuracy, precision, recall, F1 score (mean ± stdv).

	**Accuracy (%)**	**Precision (%)**	**Recall (%)**	**F1 score (%)**
Motion	91.56 ± 0.82	96.20 ± 1.23	92.12 ± 0.63	94.12 ± 0.39
Chemical shift	89.78 ± 0.66	92.86 ± 0.88	93.41 ± 1.04	93.13 ± 0.47
Radio-frequency	91.11 ± 0.89	95.27 ± 0.37	91.56 ± 0.72	93.38 ± 0.25
Normal	88.89 ± 0.21	82.81 ± 0.44	79.10 ± 1.25	80.91 ± 0.66

The proposed classification network was also compared to other state-of-the-art architectures by training/testing with the same parameters and dataset. The results of the comparison can be seen in Table 3 where RISE-Net (accuracy of 90.34% and F1 score of 90.39%) outperformed the other networks in terms of accuracy and F1-score (*p* < 0.05 for each comparison).

**Table 3:** Comparison against other classification networks (mean ± stdv).

	**Accuracy (%)**	**F1 score (%)**
RISE-Net	90.34 ± 0.65	90.39 ± 0.44
VGG-16	84.81 ± 0.46 (*p* = 2.60 ×10^−6^)	85.22 ± 0.43 (*p* = 4.10 ×10^−7^)
ResNet-50	89.05 ± 0.76 (*p* = 0.031)	87.46 ± 0.76 (*p* = 1.50 ×10^−4^)
Inception	82.30 ± 0.56 (*p* = 2.19 ×10^−7^)	78.37 ± 0.50 (*p* = 8.92 ×10^−10^)
ResNet- Inception	88.92 ± 0.33 (*p* = 0.016)	87.23 ± 0.43 (*p* = 6.59 ×10^−5^)
SE-ResNet	89.21 ± 0.35 (*p* = 0.043)	87.51 ± 0.59 (*p* = 4.69 ×10^−4^)
SE-Inception	83.02 ± 0.61 (*p* = 2.04 ×10^−6^)	78.90 ± 0.52 (*p* = 7.62 ×10^−9^)

An ablation study was also conducted where different components of the RISE-Net architecture were omitted or replaced. Specifically, one test was performed where the skip connections were completely removed. This resulted in an accuracy of 89.05% (*p* = 0.0412) and an F1 score of 88.00% (*p* = 1.08 × 10^−4^). Another test included the removal of the SE block which produced an accuracy of 83.51% (*p* = 4.38 × 10^−5^) and an F1 score of 84.41% (*p* = 8.20 × 10^−7^). Lastly, the inception layer was taken out and replaced with a single 3 × 3 convolution kernel which gave an accuracy of 89.38% (*p* = 0.0410) and an F1 score of 89.32% (*p* =0.0390). The results are also tabulated in Table 4.

**Table 4:** Ablation study for RISE-Net architecture (mean ± stdv).

	**Accuracy (%)**	**F1 score (%)**
Without skip connections	89.05 ± 0.91 (*p* = 0.041)	88.00 ± 0.42 (*p* = 1.08 × 10^−4^)
Without SE	83.51 ± 1.69 (*p* = 4.38 ×10^−5^)	84.41 ± 0.74 (*p* = 8.20 ×10^−7^)
With 3 ×3 Conv	89.38 ± 0.30 (*p* = 0.041)	89.32 ± 0.84 (*p* =0.0390)

The artifact severity network aimed to output a value between 0 and 3, where 0 represented a non-severe artifact and 3 represented a severe artifact. MSE for each class was calculated and tabulated in Table 5. MSE for the 3 artifact types motion, chemical shift, and radio-frequency were 0.097, 0.079, and 0.073, respectively. These results indicate that the predicted outputs were relatively similar to their corresponding ground truth and differed by <10%.

**Table 5:** MSE loss per class for severity regression CNN (mean ± stdv).

	**MSE**
Motion	0.097 ± 0.008
Chemical shift	0.079 ± 0.001
Radiofrequency	0.073 ± 0.001

**Figure 8:**
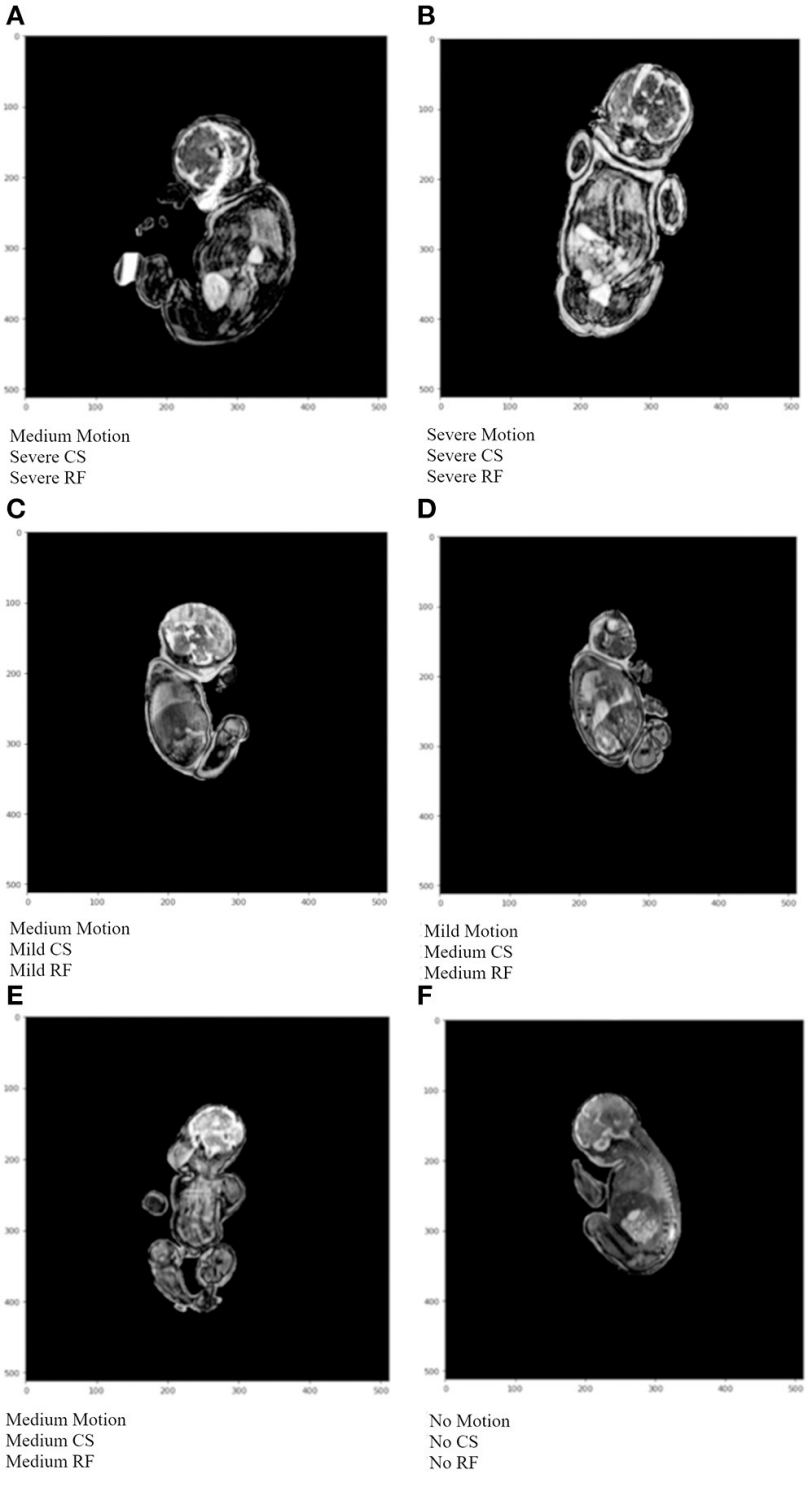
Example algorithm outputs of overall artifact severity for test images where CS represents chemical shift artifacts, and RF represents radio-frequency artifacts. In **(A)** medium motion, and severe CS and RF were detected. **(B)** shows severe artifacts for all 3 artifact types. **(C)** depicts medium motion, and mild CS and RF artifacts. In **(D)** mild motion, and medium CS and RF artifacts were detected. In **(E)** medium severity was detected for all artifact types. In **(F)** no artifacts were detected.

Examples of the final output of the algorithm are shown in Figure 8. The output from the artifact classification network was multiplied with the corresponding output from the artifact severity regression network. Based on a thresholding method, the artifacts were either labeled as containing none, mild, medium, or severe artifacts. Image A is heavily degraded by all 3 artifact types and the network output labels reflect that. Furthermore, the image has a radio-frequency artifact overlying the brain region. The network accurately detected it and output a “Severe” label for the “Radio-frequency” class. Image B is markedly degraded by artifacts. The network labeled it “Severe” for all artifact class types. In image C, the anatomy is better visible, with mild chemical shift and radio-frequency artifacts. There is moderate blurring in the body and head region, therefore outputting a “Medium” label for the “Motion” class. Image D has a thin black outline, indicating a chemical shift artifact which was labeled as “Medium.” Additionally, there are several small streaks at the top of the head region, which was labeled as “Medium” for the “Radio-frequency” class. Image E is degraded by all 3 artifact types, but less markedly than Image B leading to a “Medium” severity label for all 3 artifacts by the network. In Image E, the network detected no artifacts.

## Discussion

We introduced and evaluated a novel CNN algorithm that can automatically detect and grade the severity of artifacts in whole-body fetal MRI scans. The algorithm is a combination of 2 identical frameworks where the first is used for classification (detection of artifacts), and the second is used for regression (output severity of artifact). For the artifact classification CNN, each class was treated independently of the others since the task was multi-labeled. A confusion matrix for each class was constructed and the following metrics were calculated: accuracy, precision, recall, and F1 score. All metrics for each class were relatively close in value except for the “Normal” class. This class had significantly lower values for precision, recall, and F1 score. The main reason for this was the lack of available scans containing no artifacts at all. As a result, the “Normal” class had fewer samples, leading to a slight imbalance even though a minority class up-sampling technique was implemented. However, the other classes achieved good results in all metrics as they were able to attain values between the high-80s to mid-90s.

When averaging across the classes, RISE-Net obtained a total accuracy of 90.3% and an F1 score of 90.4%. In terms of accuracy, RISE-Net outperformed other competitive networks, including VGG-16, Res-Net 50, and Inception by 5.53% (*p* = 2.60 × 10^−6^), 1.29% (*p* = 0.031), and 8.04% (*p* = 2.19 × 10^−7^), respectively. For the F1 score, it also surpassed the same networks by 5.17% (*p* = 4.10 × 10^−7^), 2.93% (*p* = 1.50 × 10^−4^), and 12.02% (*p* = 8.92 × 10^−10^), respectively. These results demonstrate that incorporating skip connections, inception layers, and SE blocks can greatly improve performance in classification networks. Our results from the accuracy comparison are in line with those made by Rodrigues et al. who compared classification CNN architectures for immunofluorescence images (Rodrigues et al., 2020). However, the authors reported that the accuracy of ResNet-50 surpassed that of Inception, followed by VGG-16 when the same architectures were tested on the ImageNet validation set (He et al., 2015). The different findings suggest a performance dependence on the dataset used. For the comparison with other multi-model networks, RISE-Net surpassed ResNet-Inception, SE-ResNet, and SE-Inception, by 1.42% (*p* = 0.016), 1.13% (*p* = 0.043), and 7.32% (*p* = 2.04 × 10^−6^), respectively, in terms of accuracy. For F1 score, RISE-Net exceeded the same networks by 3.16% (*p* = 6.59 × 10^−5^), 2.88% (*p* = 4.69 × 10^−4^), and 11.49% (*p* = 7.62 × 10^−9^), respectively. Our findings agree with other works such as in Hu et al. (2019) where they performed experiments on ImageNet, and noted from highest ranking performance to lowest followed: ResNet-Inception, SE-ResNet, and then SE-Inception. In our case, SE-ResNet outperformed ResNet-Inception, but both of our results indicated that they had similar performances.

An ablation study was also carried out where main architecture components were taken out or replaced. In the first experiment, all skip connections were removed, which led to a decrease of 1.29% (*p* = 0.041) and 2.39% (*p* = 1.08 × 10^−4^) in accuracy and F1 score, respectively. This was expected as incorporating skip connections allowed for a deeper network to be constructed without encountering the vanishing gradient problem. While running training experiments, this held true where networks lacking skip connections stopped learning at ~ 50 epochs, where other experiments plateaued closer to 100 epochs. This was an indication that the accumulation of gradients was small and close to zero, which led to weights not being updated as training went on. Furthermore, other papers completed experiments that explored the benefits of using skip connections. Namely, Alaraimi et al. found that adding skip connections to standard models (e.g., AlexNet, VGG-16, and GoogLeNet) increased accuracy when trying to classify brain tumors in MRIs (Alaraimi et al., 2021).

The second experiment was performed with the omission of SE blocks. This change led to a decrease in accuracy of 6.83% (*p* = 4.38 × 10^−5^) and a decrease in F1 score of 5.98% (*p* = 8.20 × 10^−7^). SE blocks therefore had the greatest impact on the network. This is logical since SE blocks are used to perform feature recalibration, where a greater emphasis is placed on important features. This suggests that the network learned to isolate feature maps that directly correlated to artifacts which in turn increased accuracy. Additionally, the omission of the SE component created an Inception-ResNet hybrid model which other researchers have also performed experiments on. Specifically, Hu et al. found that there was a decrease in error of 0.42% when adding SE blocks to the original Inception-ResNet-v2 and testing on the ImageNet validation set (Hu et al., 2019).

The last experiment consisted of replacing the inception layer with a single 3 × 3 convolution filter. This was done to follow a more standard CNN architecture. The accuracy subsequently dropped by 0.96% (*p* = 0.041) and the F1 score was reduced by 1.07% (*p* = 0.039). The results indicated that there was minimal performance decrease when exchanging the components. When other researchers performed similar experiments, comparable results were reported. Taking out the inception layer essentially created a ResNet based model with SE blocks added. Several studies have reported that adding SE blocks to the base ResNet increased performance (Xu and Zhang, 2020; He and Jiang, 2021). When tested on the ImageNet validation set, there was also a decrease in error by 0.86% which is in agreement with our results (Rodrigues et al., 2020).

Overall, all components of the proposed RISE-Net seem to be necessary as removal/replacement of each one of them resulted in worsening of network performance. The most significant component of RISE-Net is its SE blocks as the performance of the network drastically decreased when it was removed. Furthermore, the inception layer had the least impact as minimal losses occurred when it was replaced with a 3 × 3 convolution kernel.

Fu et al. created a similar architecture where they adopted an Inception-ResNet-SE design. Their goal was to classify lung nodules on CT images (Fu, 2019). They were able to yield a good accuracy of 89.51% which is comparable to our results. However, their classification method was binary where they classified an image as either “benign” or “malignant.” In comparison, our algorithm utilized a multi-label approach. Our algorithm can be used to alert the operator whether an MR imaging sequence is degraded by artifacts while at the same time specifying the artifact type and severity allowing them to make an informative and objective decision about the acquired sequence. Similarly, Gagoski et al. proposed a CNN based method to classify fetal motion artifacts in HASTE sequences (Gagoski et al., 2022). In comparison to our motion class accuracy of 91.56%, they were able to achieve an accuracy of 85.2%. Their approach also solely focused on one type of artifact in the head region, while ours can detect multiple artifacts in varying anatomical locations. However, their pipeline incorporated a motion correction feature where images that were heavily degraded were automatically reacquired. Our future goals would include extending the scope of the algorithm to correct any detected artifact types to further automate and improve fetal MRI acquisition and interpretation.

## Conclusion

We present a novel fetal MR image quality assurance algorithm called RISE-Net which incorporates 2 CNNs; the first being used for artifact detection, and the second for classification of artifact type and severity. Combining the outputs from both CNNs allowed for a total artifact severity score which effectively quantifies the severity level of each artifact detected on a fetal MRI. The main mechanism for the networks was the combination of skip connections, inception layers, and SE blocks. The algorithm showed promising results as the classification network achieved an accuracy of 90.34% and an F1 score of 90.39%. The regression network across all classes had an MSE of 0.083. The algorithm has the potential to serve as a quality control tool to allow radiologists to automatically and objectively determine if a fetal MR imaging sequence is degraded by artifacts and needs to be repeated. The next steps for this project would be to incorporate it into a clinical setting and allow its impact on the clinical workflow to be studied.

## Data Availability Statement

The datasets presented in this article are not readily available because the hospital's research ethics board does not permit sharing clinical images. Requests to access the datasets should be directed to DS, dafna.sussman@ryerson.ca.

## Ethics Statement

The studies involving human participants were reviewed and approved by the Hospital for Sick Children and Toronto Metropolitan University (formerly Ryerson University). Written informed consent for participation was not required for this study in accordance with the national legislation and the institutional requirements.

## Author Contributions

AL and DS created the methods to the project. AL and JL carried out all experiments. Manuscript was written by AL, JL, and DS. Fetal MRI dataset, corresponding labels, and clinical validation were provided by BE-W and MW. DS funded the project. All authors have seen and edited the manuscript. All authors contributed to the article and approved the submitted version.

## Funding

NSERC-Discovery Grant RGPIN-2018-04155 (Sussman) provided funding for the study.

## Conflict of Interest

The authors declare that the research was conducted in the absence of any commercial or financial relationships that could be construed as a potential conflict of interest.

## Publisher's Note

All claims expressed in this article are solely those of the authors and do not necessarily represent those of their affiliated organizations, or those of the publisher, the editors and the reviewers. Any product that may be evaluated in this article, or claim that may be made by its manufacturer, is not guaranteed or endorsed by the publisher.
